# SPIN90 Knockdown Attenuates the Formation and Movement of Endosomal Vesicles in the Early Stages of Epidermal Growth Factor Receptor Endocytosis

**DOI:** 10.1371/journal.pone.0082610

**Published:** 2013-12-10

**Authors:** Hyejin Oh, Hwan Kim, Kyung-Hwun Chung, Nan Hyung Hong, Baehyun Shin, Woo Jin Park, Youngsoo Jun, Sangmyung Rhee, Woo Keun Song

**Affiliations:** 1 Bio Imaging and Cell Dynamics Research Center, School of Life Sciences, Gwangju Institute of Science and Technology (GIST), Cheomdan Gwagi-ro 261, Gwangju Metrocity, Korea; 2 Bio Remodeling and Gene Therapy Laboratory, School of Life Sciences, Gwangju Institute of Science and Technology (GIST), Cheomdan Gwagi-ro 261, Gwangju Metrocity, Korea; 3 Cell Biology and Membrane Biochemistry Laboratory, School of Life Sciences, Gwangju Institute of Science and Technology (GIST), Cheomdan Gwagi-ro 261, Gwangju Metrocity, Korea; 4 Department of Cancer Biology, Vanderbilt University Medical Center, Nashville, Tennessee, United States of America; 5 Department of Life Science, Chung-Ang University, Seoul, Korea; 6 Center for Human Genetic Research, Massachusetts General Hospital, Harvard Medical School, Boston, Massachusetts, United States of America; Hungarian Academy of Sciences, Hungary

## Abstract

The finding that SPIN90 colocalizes with epidermal growth factor (EGF) in EEA1-positive endosomes prompted us to investigate the role of SPIN90 in endocytosis of the EGF receptor (EGFR). In the present study, we demonstrated that SPIN90 participates in the early stages of endocytosis, including vesicle formation and trafficking. Stable HeLa cells with knockdown of SPIN90 displayed significantly higher levels of surface EGFR than control cells. Analysis of the abundance and cellular distribution of EGFR via electron microscopy revealed that SPIN90 knockdown cells contain residual EGFR at cell membranes and fewer EGFR-containing endosomes, both features that reflect reduced endosome formation. The delayed early endosomal targeting capacity of SPIN90 knockdown cells led to increased EGFR stability, consistent with the observed accumulation of EGFR at the membrane. Small endosome sizes and reduced endosome formation in SPIN90 knockdown cells, observed using fluorescent confocal microscopy, strongly supported the involvement of SPIN90 in endocytosis of EGFR. Overexpression of SPIN90 variants, particularly the SH3, PRD, and CC (positions 643 - 722) domains, resulted in aberrant morphology of Rab5-positive endosomes (detected as small spots located near the cell membrane) and defects in endosomal movement. These findings clearly suggest that SPIN90 participates in the formation and movement of endosomes. Consistent with this, SPIN90 knockdown enhanced cell proliferation. The delay in EGFR endocytosis effectively increased the levels of endosomal EGFR, which triggered activation of ERK1/2 and cell proliferation via upregulation of cyclin D1. Collectively, our findings suggest that SPIN90 contributes to the formation and movement of endosomal vesicles, and modulates the stability of EGFR protein, which affects cell cycle progression via regulation of the activities of downstream proteins, such as ERK1/2, after EGF stimulation.

## Introduction

Endocytosis is the process by which cells take up extracellular macromolecules through vesicles from their environment, and encompasses pinocytosis, phagocytosis, and clathrin/caveolae-dependent endocytosis. This process regulates a variety of cellular functions, and contributes, at least in part, to important aspects of cell physiology, such as cellular adhesion and migration [1], [2], drug delivery [3], receptor downregulation [4], [5] and tissue homeostasis [6].

Studies on the epidermal growth factor receptor (EGFR) in chick embryo back skin, which proliferates rapidly in EGF-containing medium, highlight the importance of EGF for cell proliferation and cancer development [7]. The roles of EGFR, a receptor tyrosine kinase, in epithelial development are further reflected by defects in eye formation, skin (hair follicle and epidermis), and intestinal villi of EGFR knockout mice [8], [9]. The hepatitis B virus (HBV), the primary cause of hepatocellular carcinoma, upregulates EGFR expression and disrupts the fine tuning of EGFR-mediated signal transduction [10]. These findings support critical roles of EGFR in differentiation, pathogenesis, and cell survival.

Six ligands of EGFR, specifically, transforming growth factor- α(TGF-α), amphiregulin, heparin-binding EGF-like growth factor (HB-EGF), betacellulin, epiregulin, and EGF [11], [12], evoke different intracellular responses. In resting cells, empty EGFR (without ligand) is usually recycled back to the plasma membrane, whereas ligand-bound EGFR is activated through auto-phosphorylation to provide platforms for interactions with signaling proteins and endocytic regulators. For example, phosphorylation at Tyr-920 controls PI3K/Akt signaling through regulation of interactions of EGFR with p85, while phosphorylation at Tyr-1068 stimulates binding of EGFR with Grb2, which can trigger Ras/mitogen-activated protein (MAPK) signaling [13]. These interactions facilitate internalization of EGFR into the cytoplasm via vesicle formation.

Association of EGFR with endosomal components transduces the activated receptor signal to downstream targets, which is downregulated by receptor degradation within the late endosome/lysosome compartment. Receptor activation requires several steps controlled by endocytic machineries, such as clathrin, dynamin, syndapin, and Rab GTPases, including ligand-induced internalization from the plasma membrane through vesicle formation and delivery into the internal endosomes of destination. In particular, the endosomal compartment acts as an intermediate in signaling between the plasma membrane and nucleus [14], and both spatial and temporal regulation of endocytosis are critical for maintenance of homeostasis in cell physiology [15], [16].

Analysis of growth factor-induced signal transduction that involves cell cycle machinery [17], [18] has revealed that increased activation of downstream proteins drives resting cells into the S phase [19] through increased abundance and/or activities of cell cycle regulators, such as cyclin D, cyclin E, and cyclin-dependent kinases [20], [21]. Cell proliferation is a major physiological outcome of EGFR activation. Overexpression of EGFR causes hyper-proliferation, both *in vitro* and *in vivo,* and upregulation of EGFR is observed in a number of malignant cancers. This event is caused by hyperactivation of various downstream targets in EGFR signaling, such as the serine/threonine kinase, Raf, and MAPK/extracellular signal-regulated kinase 1/2 (ERK1/2) [22]. Therefore, defects in this endocytic route may result in accumulation of activated EGFR, which sustains mitogenic signaling and results in aberrant proliferation. Elucidation of the mechanisms involved in defective endocytosis and failure of receptor downregulation should provide insights into the mechanisms involved in tumor proliferation.

SPIN90, a Nck-binding protein, is known to regulate actin polymerization. Earlier studies by our group showed that SPIN90 PRD in Cos7 cells is associated with syndapin I, which is required for CCV (Clathrin Coated Vesicle) formation [23], and SPIN90 SH3 binds with dynamin I to catalyze the budding of vesicles from the plasma membrane [24] in neuronal cells. In the current study, we present evidence that SPIN90 participates in EGFR endocytosis. SPIN90 knockdown caused a delay in EGFR endocytosis, whereby the majority of EGFR was detected on the cell surface, and not within the cell. This finding may attributed to endocytic defects, such as early endosome targeting and vesicle/endosome formation. Overexpression of SPIN90 variants led to alterations in the morphology and movement of early endosomes. Finally, knockdown of SPIN90 increased the stability of EGFR, in turn, affecting cell cycle progression by sustaining the activities of downstream proteins, such as ERK1/2, after EGF stimulation.

## Materials and Methods

### Antibodies and reagents

Rabbit anti-SPIN90 polyclonal serum has been described previously [25]. Rabbit polyclonal antibodies were used to detect EGFR and phospho-EGFR (Santa Cruz Biotechnology, Texas, USA). Mouse monoclonal antibodies used against phospho-ERK and ERK were obtained from Cell Signaling (Denver, USA), and anti-EGFR (528) monoclonal antibody from Santa Cruz Biotechnology. Goat polyclonal antibody against the early endosome marker, designated early endosome antigen 1 (EEA1), was acquired from Santa Cruz Biotechnology. Horseradish peroxidase-conjugated anti-mouse, anti-rabbit and anti-goat secondary antibodies were purchased from the Jackson Laboratory (Maine, USA). Alexa Fluor 488-, Alexa Fluor 555-, Alexa Fluor 594-, and Alexa Fluor 647-conjugated donkey anti-rabbit and donkey anti-mouse IgG were from Molecular Probes (Invitrogen, Carlsbad, CA, USA). PD98059, an inhibitor of MEK1/2, was acquired from Selleck (Houston, TX, USA).

### Plasmids and primers

SPIN90 cDNA was inserted into pEGFP-c1 or pcDNA 3.0-HA vector (Clontech). The pECFP-c1-Rab5a construct was kindly provided by Dr. M. Zerial (Max Plank Institute, Germany). Specific oligomers of EGFR and SPIN90 for RT-PCR were: sense, 5′-CAGGGACCGCGAGAACCACA-3′, and antisense, 5′-CCTCCAC-TGGATAGTATC-3′; sense, 5′-ACTGTGCGGCACATCG-3′, and antisense, 5′-GCTGGGAGCCTCCC-CCAG-3′, respectively. SPIN90 SH3 (amino acids [a.a.] 1 - 57), PRD (a.a. 58 - 279), N-term (a.a. 1 - 279), C-term (a.a. 280 - 722), CN (a.a. 280 - 462), CM (a.a. 463 - 642), and CC (a.a. 643 - 722) fragments were subcloned into the pEGFP-c1 vector.

### Cell culture, transfection, and immunocytochemistry

To generate a stable SPIN90 knockdown HeLa cell line, the si-SPIN90 nucleotides were inserted into pLKO.1-puro plasmid for lentiviral packaging using the Mission RNAi system (Sigma). HeLa cells were treated with hexadimethrine bromide after 20 h of plating. Pre-mixed lentiviral particles containing the SPIN90 shRNA construct (pLKO.1-puro-si-SPIN90) were applied to cells, followed by culture for 20 h. The medium was exchanged with fresh culture medium containing puromycin (2 µg/ml) for selection, according to the manufacturer’s instructions. Five selected colonies were verified with immunoblot analysis using the SPIN90 antibody. Among five siRNA sequences, siRNA#1-CCGGCCAGCTGTATTCTTTGCCTTACTCGAGTAAGG CAAAGAATAGAGCTGGTTTTTTG, was selected, since it suppressed SPIN90 expression with ∼80% efficiency. SPIN90 knockdown cells were maintained in DMEM supplemented with 10% FBS and puromycin (2 µg/ml) (high glucose). The morphologies and movements of Rab5-positive endosomes were monitored by transfecting HeLa cells using Lipofectamine^TM^ LTX supplemented with PLUS reagent (Invitrogen). Cells were plated on 18 mm wide coverslips at a density of 1×10^5^ cells/60 mm plastic dish. Following transfection, cells were cultured for 24 h, followed by serum starvation for 16 h. For live-cell imaging of cells overexpressing SPIN90 variants, the culture medium was replaced with Live Cell Imaging Solution (Invitrogen) containing Alexa Fluor 647 conjugated-EGF (40 ng/ml), and the plate placed within a chamber for 5 min before image acquisition. The temperatures of the chamber body, objective lens and chamber lid were maintained at 37°C, 39°C, and 40°C, respectively. Control cells were cultured on 12mm coverslips and transfected with siRNA-hH1zeo-G2 vector or shRNA-hH1zeo-G2-SPIN90-1731 (si-1731) after 24 h. After transfection, cells were cultured for 20 h, followed by serum starvation for 16 h. Cells were incubated for 30 min on ice to allow TR-EGF (40 ng/ml) binding to surface EGFR followed by incubation at 37°C for 10 min to allow EGFR internalization into the cytoplasm. For immunocytochemistry, cells were fixed with 4% paraformaldehyde in phosphate-buffered saline (PBS) containing 1 mM CaCl_2_ and 1 mM MgCl_2_, and permeabilized via incubation in 0.5% Triton X-100 for 5 min. Cells were blocked for non-specific antibody recognition with 2% BSA/PBS, and stained with the indicated antibodies for 30 min at 37°C. Images were acquired with a FV1000 confocal microscope (Olympus, JAPAN).

### Measurement of endosome morphology and EGF trafficking to early endosomes

Morphometric measurements were made using MetaMorph software. Each experiment was performed on two or three independent coverslips, and usually three times with independent cell cultures. In most cases, cells were treated with TR-EGF or EGF conjugated to Alexa Fluor-647 to stimulate EGFR endocytosis and visualize EGFR-containing vesicles. To determine endosome size, 300 – 500 endosomes (from 2 – 6 cells) were measured for each condition. Endosome size was measured as the width of EGF- containing endosomes that also included either EEA1 or Rab5. Images were acquired with a FV1000 confocal microscope. The percentage of colocalized signals within a single cell was integrated using the colocalization measurement function of Metamorph software, and regression analysis performed for statistical evaluation of differences. All images were acquired using identical acquisition parameters. Data are presented as means ± standard error of the mean (SEM). Significance was determined using paired or unpaired Student's *t*-test. Values of *p* < 0.05 were considered significant.

### Quantification of surface EGFR via immunoprecipitation

Control and SPIN90 knockdown HeLa cells were plated on 100 mm culture dishes and cultured for 24 h. Sub-confluent dishes of cells were serum-starved in serum-free DMEM at 37°C for 16 h. Cells were washed twice with ice-cold PBS, and the medium exchanged with serum-free medium containing 2 µg/ml monoclonal anti-EGFR antibody (528). Samples were incubated for 30 min on ice with shaking to allow anti-EGFR (528) antibody to detect surface EGFR. Cells were washed twice with ice-cold PBS and solubilized with lysis buffer (20 mM Tris-HCl, pH 8.0, 150 mM NaCl, 1% Triton X-100, 5 mM sodium vanadate, 50 mM sodium fluoride, 10 mM sodium pyrophosphate, and proteinase inhibitors). Protein concentrations were determined using the Bradford assay (Bio-Rad Laboratories). Cell extracts were incubated with protein A/G-Sepharose beads (GE Healthcare) for 3 h and precipitated. To determine SPIN90 interaction with EGFR, HeLa cells were solubilized with RIPA buffer (50 mM Tris-HCl, pH 8.0, 150 mM NaCl, 1% Triton X-100, 1% NP-40, 5 mM sodium vanadate, 50 mM sodium fluoride, 10 mM sodium pyrophosphate, and proteinase inhibitors) and clarified by centrifugation. Cell extracts were incubated with rabbit anti-IgG (rabbit) and rabbit anti-SPIN90 antibodies for 4 h prior to addition of protein A/G-Sepharose beads for 3 h. Immunoprecipitates were washed extensively with extraction buffer, separated using SDS-PAGE, and transferred to PVDF membranes. After blocking with 5% BSA or 5% non-fat milk in 0.1% Tween-20 containing Tris-buffered saline (TBS), the membranes were probed with primary antibodies, followed by horseradish peroxidase-conjugated secondary antibody. Blots were detected with enhanced chemiluminescence reagent (Dogen, Korea).

### Electron microscopy

For EM analysis, anti-mouse antibody conjugated to 25 nm colloidal gold particles was used to detect anti-EGFR monoclonal antibody. Both control and SPIN90 knockdown cells were fixed by incubation in 4% paraformaldehyde in PBS with rotation for 1 h at 4°C. Cells were dehydrated thorough a graded ethanol series, exchanged with propylene oxide, and embedded in Epon 812. Thin sections (80 nm) were cut with a diamond knife and collected on bare 150-mesh nickel grids, and immunogold stained [26]. Images were captured under a FEI TECNAI G^2^ transmission electron microscope operating at 120 kV.

## Results

### SPIN90 participates in EGFR endocytosis

**Figure 1:**
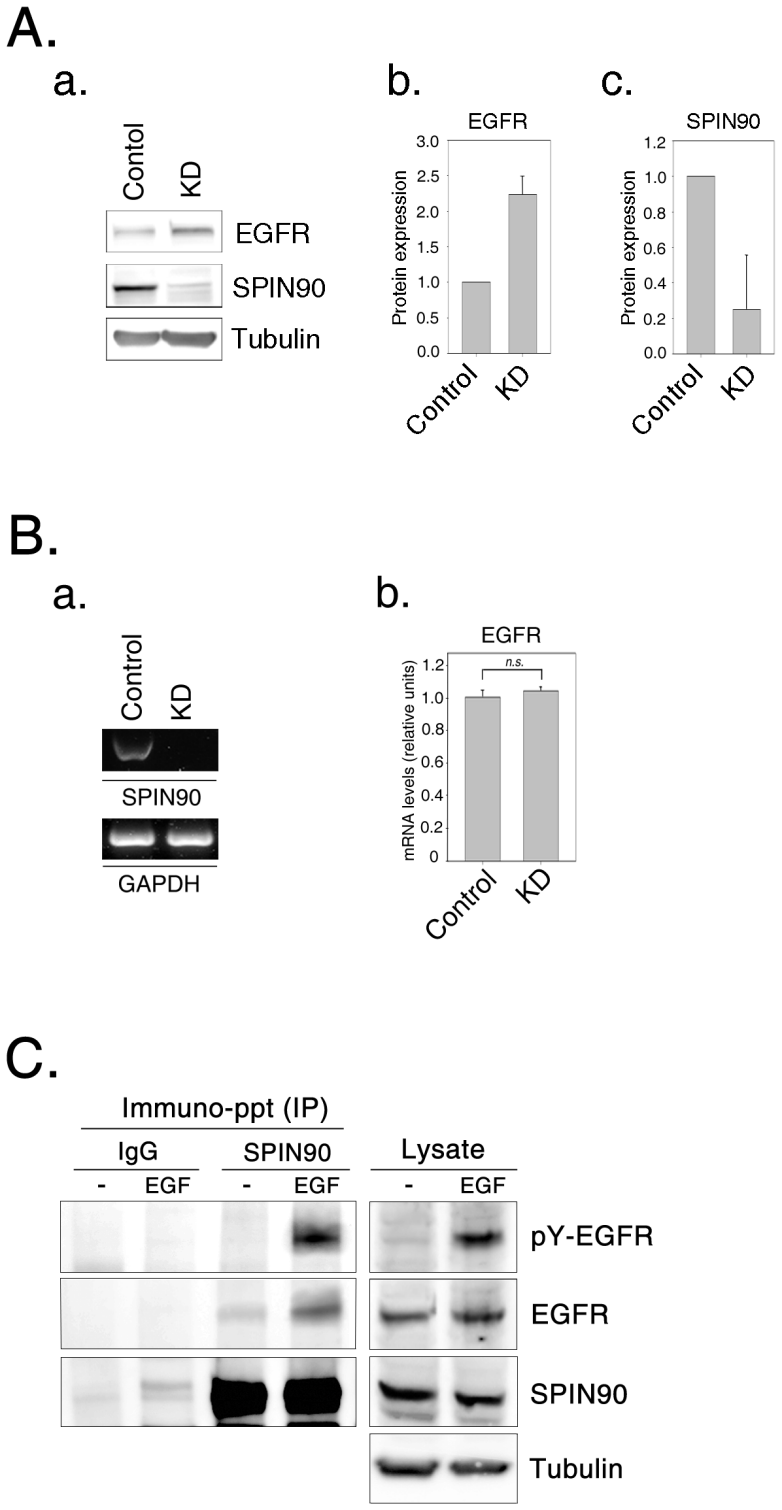
SPIN90 is a specific regulator of EGFR endocytosis. **A.** (a) Whole-cell lysates of control and SPIN90 knockdown (KD) HeLa cells were separated using SDS-PAGE, and immunoblotted with polyclonal anti-EGFR antibody to quantify the total amounts of EGFR. The experiment was repeated more than three times. Levels of EGFR (b) and SPIN90 (c) were quantified via densitometry using MultiGauge software. **B.** The relative abundance of SPIN90 (a) and *EGFR* mRNA (b) was quantified in control and SPIN90 knockdown (KD) HeLa cells using RT-PCR (a) or qPCR (b). Values of qPCR for *EGFR* transcripts were normalized with those for *GAPDH* transcripts. **C.** Identification of the SPIN90 interaction with EGFR. Cells were subject to starvation for 16 h then treated with EGF (40 ng/ml) for 10 min. Cell lysate were immunoprecipitated with rabbit anti-IgG or rabbit anti-SPIN90 antibodies and blotted with rabbit anti-pY-EGFR antibody.

**Figure 2:**
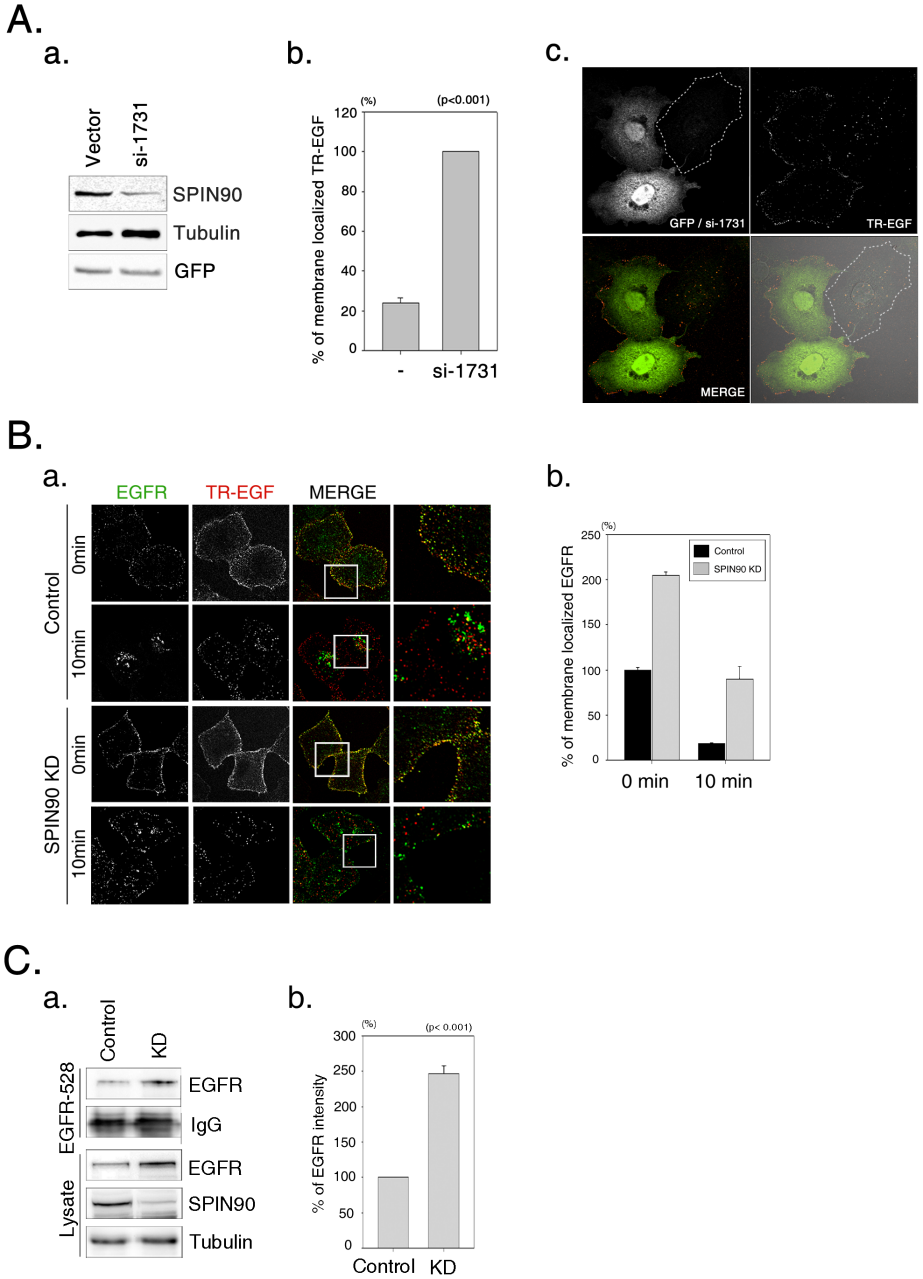
SPIN90 knockdown increases the levels of surface EGFR. **A.** HeLa cells were transiently transfected with either siRNA-hH1zeo-G2 vector or siRNA-hH1zeo-G2-SPIN90 1731 (si-1731) for knockdown of SPIN90. (**a**) SPIN90 knockdown was verified by immunoblotting using an anti-SPIN90 antibody. (**b**) Graphs indicate the levels of TR-EGF localized at membrane in si-1731 transfected cells and non-transfected cells. (**c**) siRNA-hHazeo-G2-SPIN90 1731 transfected cells were visualized by green fluorescent proteins (green). An untransfected control cell is marked by a dotted line. **B.** (a) Control and SPIN90 knockdown (KD) HeLa cells were treated with 40 ng/ml Texas Red (TR)-EGF for 30 min at 4°C, followed by additional incubation for 0 or 10 min. Cells were fixed and stained with polyclonal anti-EGFR antibody and FITC secondary antibody. (b) The fluorescence intensity of membrane EGFR labeled with TR-EGF was quantified using Metamorph software. **C.** Cells were starved for 16 h and stained with monoclonal anti-EGFR (528) antibody for 30 min at 4°C. Cell lysates were incubated with protein A/G Sepharose beads for 2 h, and resolved using SDS-PAGE. (a) Surface EGFR was blotted with anti- EGFR antibody. (b) is the surface EGFR-quantified result of (a).

The observed colocalization of SPIN90 with EEA and EGF in early endosomes led to the speculation that SPIN90 is functionally associated with EGFR endocytosis. Stable HeLa cells depleted of SPIN90 (SPIN90 KD) or transfected with vector (control) were generated using the lentiviral system. SPIN90 expression was suppressed with an efficiency of ∼80% upon SPIN90 knockdown. Notably, SPIN90 knockdown was associated with a substantial increase in the total amount of EGFR in cell lysates (Fig. 1A). However, the EGFR mRNA level was not significantly different between control and SPIN90 knockdown cells (Fig. 1B). In addition, endogenous SPIN90 was coimmunoprecipitated with EGFR after EGF stimulation; interestingly, phosphorylated EGFR strongly interacted with SPIN90 (Fig. 1C), suggesting that SPIN90 participates in the regulation of EGFR endocytosis. In view of this finding, we questioned whether endocytic effects contribute to the increased EGFR level. Consequently, internalization of Texas Red-EGF (TR-EGF) and surface EGFR level were assessed. First, we transfected cells with siRNA-hH1zeo-G2-SPIN90-1731 construct (si-1731) for SPIN90 knockdown (Kim et al., 2006) and measured EGFR internalization. At 10 min after TR-EGF stimulation, most TR-EGF was internalized in non-transfected cells whereas 80% of TR-EGF remained at the membrane in si-1731 transfected cells, indicating that EGFR internalization was dramatically inhibited (Fig. 2A). Same experiments were performed with stable SPIN90 knockdown cell lines. Higher levels of EGFR remained at the membrane (Fig. 2B) in SPIN90 knockdown cells, compared to control cells. Immunoblot analysis of EGFR located on the cell surface using anti-EGFR (528) monoclonal antibody additionally revealed significantly higher levels of surface EGFR in SPIN90 knockdown than control cells (Fig. 2C).

**Figure 3:**
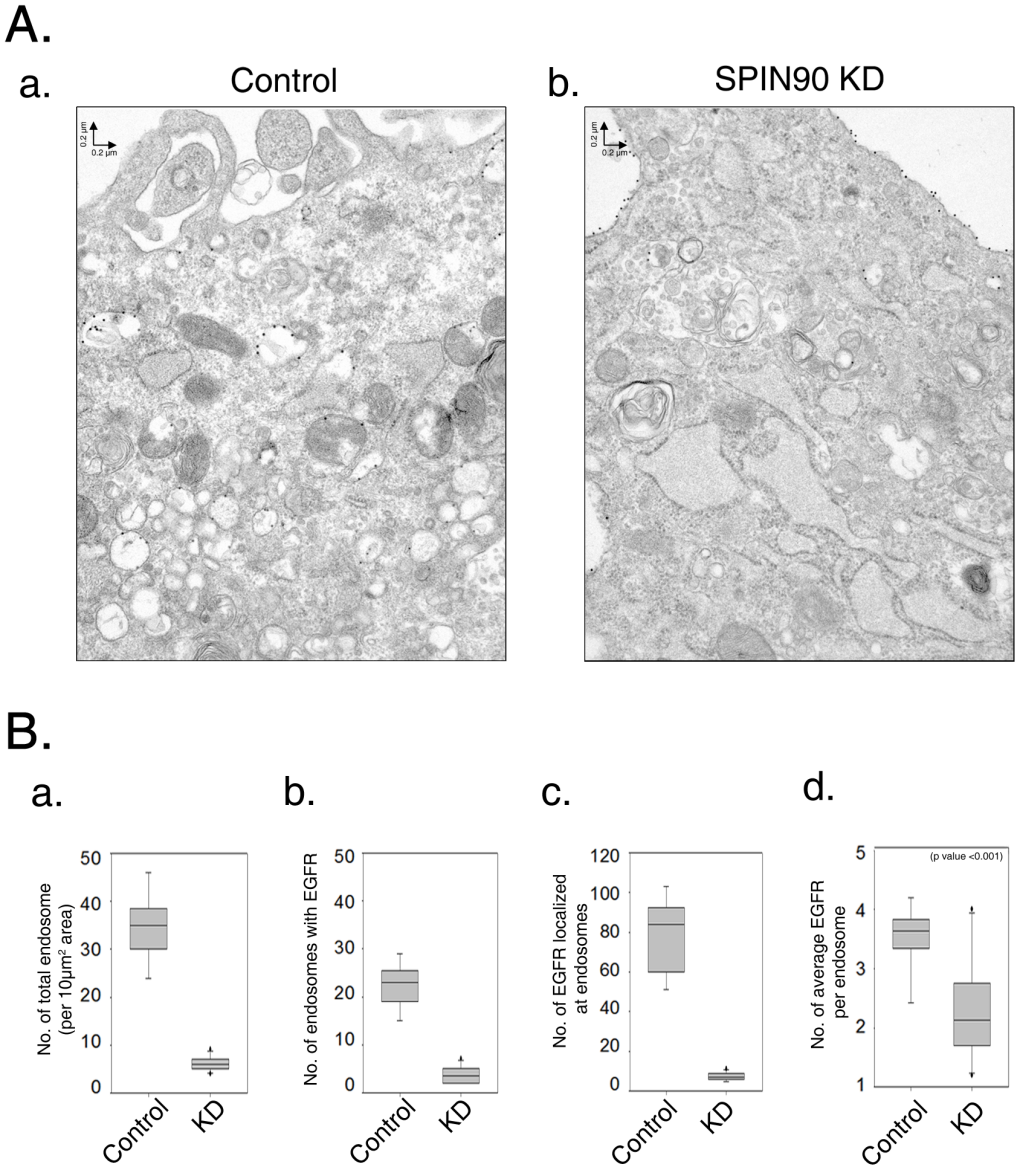
Immunogold staining of EGFR using electron microscopy. **A.** Control and SPIN90 knockdown (KD) HeLa cells were cultured for 24 h and suspended in serum-free medium for an additional 1 h. Cells treated with 40 ng/ml EGF were incubated for 10 min to allow EGFR internalization, and fixed with 4% paraformaldehyde. Cells were prepared for electron microscopy, as described in Material and Methods. Sectioned samples were stained with mouse anti-EGFR antibody and labeled with 25 nm gold particles conjugated to anti-mouse antibody. B**.** Numbers of total endosomes per image (4.8 µm×6.0 µm) (a), numbers of endosomes with EGFR (b), numbers of EGFR localized at endosomes (c) and average EGFR numbers per endosome (d) were analyzed.

To further ascertain the endocytic defects of EGFR, internalization was analyzed with immunogold staining followed by examination under a transmission electron microscope (TEM; Fig. 3). Immunogold staining of EGFR revealed that the majority of EGFR is present in endosomes of control cells within 10 min after EGF stimulation. However, most EGFR in SPIN90 knockdown cells remained at the membrane (Fig. 3A). Moreover, the number of total endosomes and EGF-containing endosomes was notably reduced in SPIN90 knockdown cells (Fig 3B). Our data showing enhanced surface EGFR in SPIN90 knockdown cells clearly indicate that SPIN90 participates in EGFR endocytosis

### SPIN90 affects vesicle formation in the early stages of endocytosis

**Figure 4:**
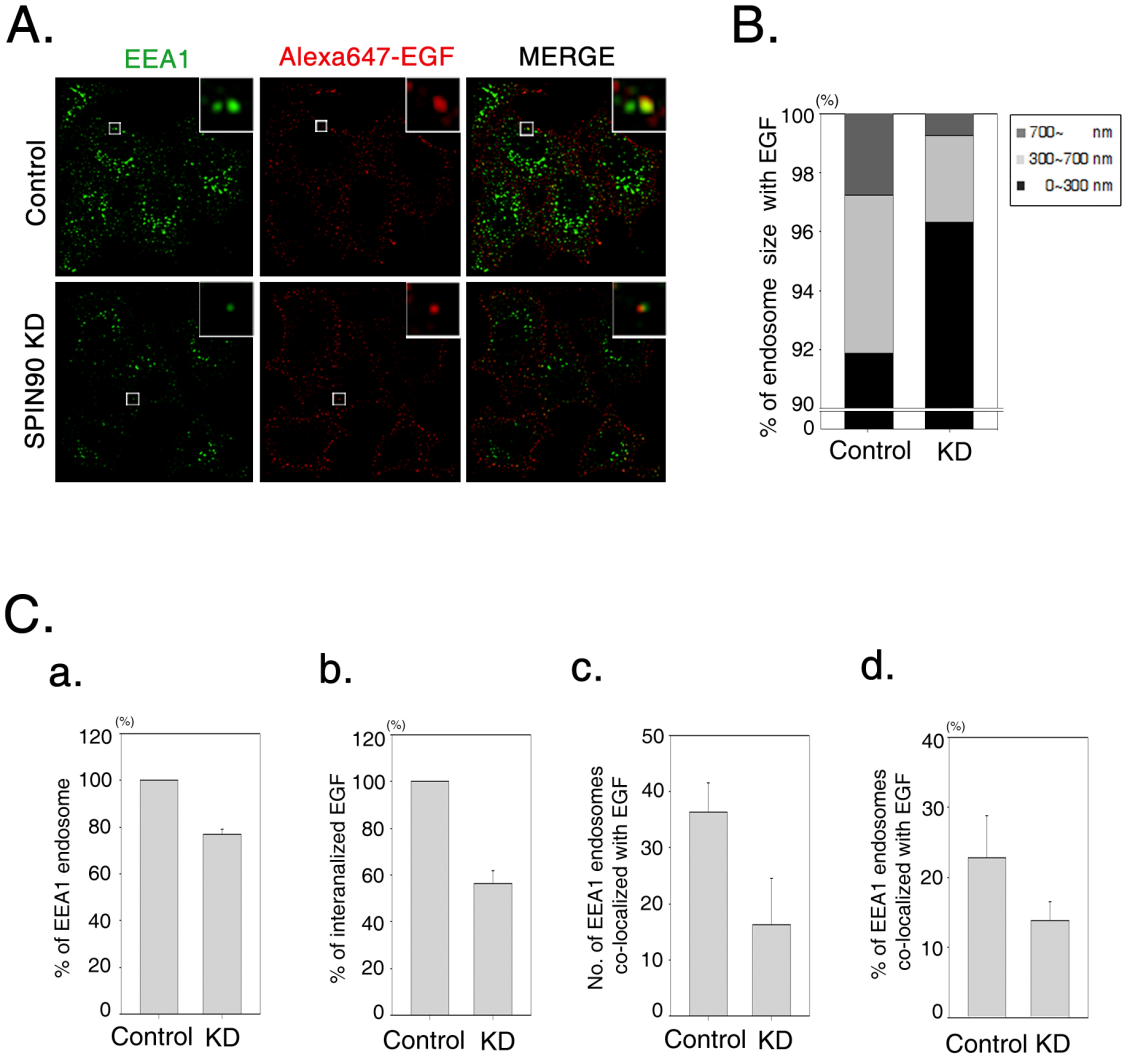
SPIN90 knockdown suppresses the formation of EEA1-positive endosomes. **A.** Cells were treated with Alexa Fluor 647-conjugated EGF (40 ng/ml) for 10 min and fixed, prior to immunostaining with anti-EEA1 antibody. **B.** Bar graphs were generated using Sigmaplot. In total, 120 – 170 endosomes per cell were measured using three criteria (vesicle width: 0 – 300 nm, 300 – 700 nm and >700) in 60 control and SPIN90 knockdown cells (KD). Overall, 150.70±5.82 vesicles (91.89%) in control cells and 121.36±7.65 (96.32%) vesicles in SPIN90 knockdown cells (KD) were 0 – 300 nm in width; 8.77±2.34 (5.35%) in control cells, 3.69±0.16 (2.93%) in KD cells were 300 – 700 nm in width; 4.53±0.67 (2.76%) in control cells, 0.93±0.16 (0.74%) in KD cells were over 700 nm in width. **C.** Total numbers of EEA1 endosomes (a), internalized EGF (b), and numbers of EEA1-endosomes containing EGF (c) were analyzed in control and SPIN90 knockdown HeLa cells. All values were determined using Metamorph Software.

Next, we analyzed endosome formation in both SPIN90 knockdown and control cells (Fig. 4). First, the sizes (measured based on endosome width) of EGF-containing vesicles that colocalized with EEA1, an early endosome marker, were assessed using three criteria (endosome width; 0 – 300 nm, 300 – 700 nm and >700 nm). In control cells, most vesicles (91.89%) were 0–300 nm, 5.35% of vesicles had a width of 300 – 700 nm and 2.76% of vesicles were over 700 nm in width. In SPIN90 knockdown cells, the number of small-sized vesicles (0 – 300 nm) was increased (+ 4.43%), middle-sized vesicles (300 – 700 nm) decreased (– 2.42%), and large vesicles (>700 nm) decreased (–2.02%) (Fig.3B). In addition, vesicles containing both Alexa Fluor 647-EGF and EEA1 displayed a larger spot in control cells than SPIN90 knockdown cells, indicating that EGF-containing vesicles are fused with EEA1-positive early endosomes (Fig. 4A). However, the sizes of EEA1-positive endosomes fused with EGF and their total numbers (Fig. 4C) were reduced in SPIN90 knockdown cells, indicating that SPIN90 is actively involved in vesicle formation at the early stages of EGFR endocytosis. In addition, whereas EGFR in control cells was extensively internalized into endosomes and endosomal EGFR was detected far from the cell surface, only a few EGFR-containing endosomes were observed near the cell membrane in SPIN90 knockdown cells, consistent with immunogold staining data (Fig. 3). Our data collectively indicate that knockdown of SPIN90 retards the targeting ability of vesicles to the early endosome.

### SPIN90 variants alter the morphology and movement of EGF-containing vesicles

**Figure 5:**
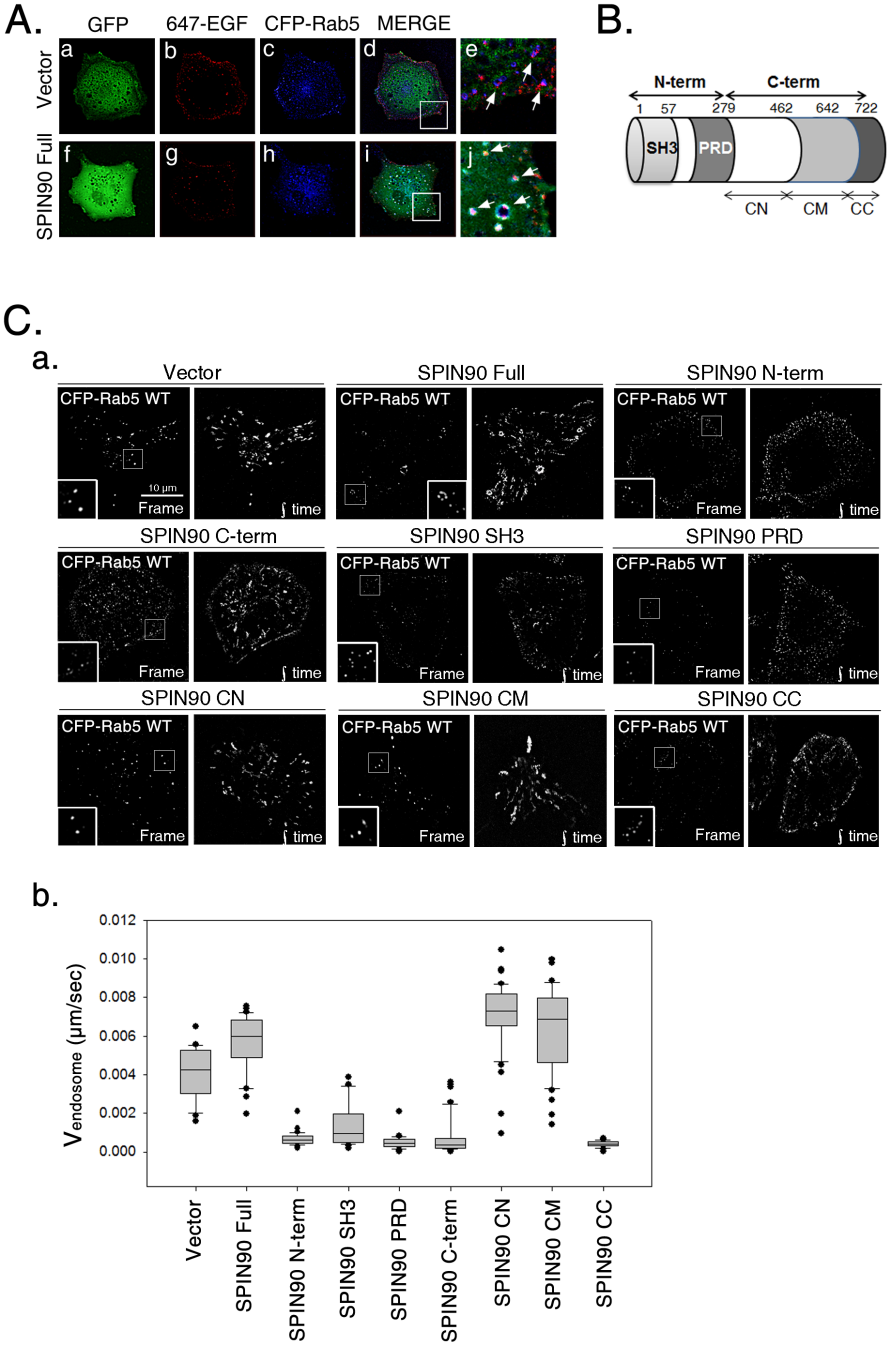
Overexpression of SPIN90 variants alters the morphology and movement of early endosomes. **A.** HeLa cells transfected with CFP-tagged Rab5 WT and full-length SPIN90 or empty vector were starved for 16 h, treated with Alexa Fluor 647-conjugated EGF (40 ng/ml), and live images acquired. **B.** Schematic diagram of SPIN90 representing functional domains. The SPIN90 N-terminal region includes SH3 and PRD (Proline-rich Domain), while the C-terminus is divided into three sections: CN, CM, and CC. **C**. (a) Moving images of CFP-Rab5-positive vesicles were acquired every 5 sec for 5 min, and the frames merged into one image to trace the movement of Rab5-positive vesicles. (b) The total lengths of the moving paths were measured and divided into 300 sec to determine the velocity of vesicles using Metamorph Software.

In view of the reduced vesicle formation during EGFR endocytosis in SPIN90 knockdown cells, we aimed to identify the functional domains of SPIN90 that affect EGFR endocytosis. CFP-Rab5 wild-type (WT), an early endosome marker [28], was used to visualize the early endosome. Initially, SPIN90 was co-expressed in HeLa cells with CFP-Rab5 wild-type (WT) to examine the morphology of vesicles containing both proteins (Fig. 5A). Overexpressed GFP was distributed evenly throughout the cytoplasm and Rab5-positive vesicles appeared as a spot-like pattern (Fig. 5A a-e). However, overexpression of full-length SPIN90 manifested as enlarged Rab5-positive vesicles with Alexa Fluor 647-EGF (Fig. 5A, f-j). Vesicle movement was additionally examined in HeLa cells overexpressing SPIN90 variants (Fig. 5B and C). To trace vesicle movement, fluorescence images were captured from vesicles labeled with CFP-Rab5 WT every 5 sec for 5 min. Overexpression of full-length SPIN90 resulted in an enlarged, doughnut-like ring morphology and more extensive movement of vesicle (velocity, 0.0057±0.0003 µm), compared to that in vector-expressing (control) cells (velocity, 0.0041±0.0003 µm) over the same period. Overexpression of the SPIN90 N-terminus, including SH3 and PRD (Proline-rich domain) domains, resulted in small, spot-like vesicles near the cell surface, indicating abnormalities in vesicle formation and trafficking (velocity of SH3, 0.0014±0.0002 µm; velocity of PRD, 0.0005±0.0001 µm). We speculate that these aberrations are possibly caused by lack of SPIN90 interactions with dynamin I and/or syndapin I through its SH3 and/or PRD [27], [29]. Interestingly, overexpression of the SPIN90 C-terminus induced retention of Rab5-positive vesicles near the plasma membrane (velocity, 0.0008±0.0001 µm), which resembled those observed in cells overexpressing SPIN90 N-terminus. Experiments with cells overexpressing the CN, CM, and CC fragments revealed that SPIN90 CC, but not CN and CM, induces defective endocytosis of EGFR (velocity, 0.0004±0.0001 µm), similar to that observed with overexpression of the SPIN90 N-terminus. Accordingly, we conclude that the SPIN90 CC domain participates in endosome formation and trafficking during EGFR endocytosis.

### SPIN90 knockdown enhances cell proliferation

**Figure 6:**
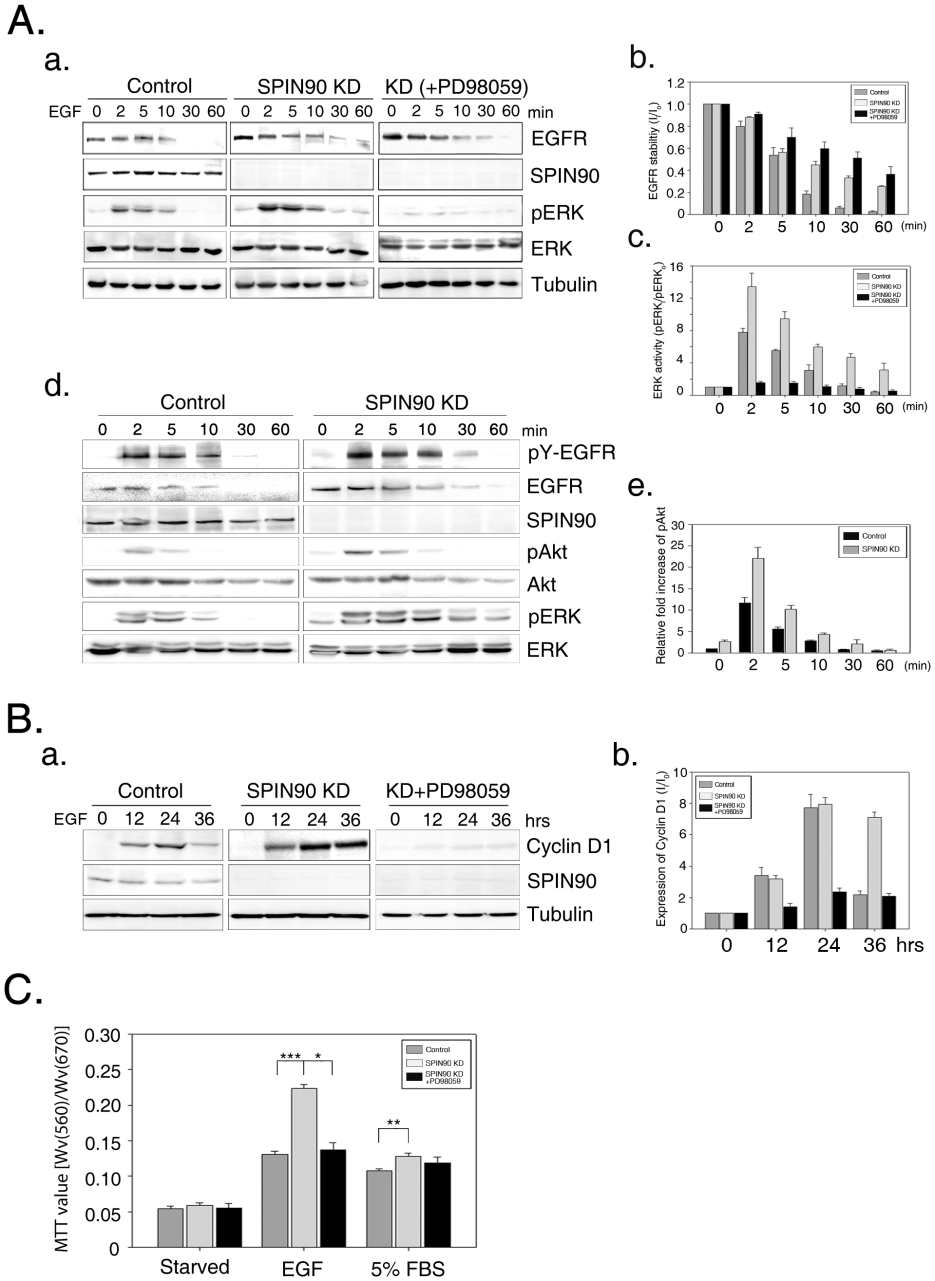
SPIN90 knockdown enhances cell proliferation. **A.** (a) Control and SPIN90 knockdown (KD) HeLa cells were starved for 16 h, and treated with 40 ng/ml EGF for the indicated times. One set of SPIN90 knockdown cells was treated with 40 ng/ml EGF and 50 µM PD98059 for the indicated times (KD+PD98059). Cell lysates were immunoblotted with the indicated antibodies. (b) Band intensities was normalized to that of tubulin. Bar graphs represent EGFR stability, calculated as the ratio of band intensity of EGFR at the indicated time-points (Ii), compared to that at 0 time (Io). (c) ERK activity is presented as the ratio of band intensity of phospho-ERK (pERK), compared to that of ERK. (d) Experiments were performed the same as Fig. 6A. Cell lysates were immunoblotted with the indicated antibodies. (e) Bar graphs represent active Akt, calculated as the ratio of band intensity of phosphor-Akt (pAkt) compared to total Akt.. **B.** Control, SPIN90 knockdown (KD) and PD98059 (50 µM)-treated SPIN90 KD HeLa cells starved for 16 h were incubated with 40 ng/ml EGF for the indicated times. PD98059 (50 µM) was used. Cyclin D1 was detected via immunoblot analysis, and the relative values of band intensities calculated. **C**. Control, SPIN90 knockdown (KD) and PD98059 (50 µM)-treated SPIN90 KD HeLa cells were incubated with 40 ng/ml EGF or 5% FBS for 36 h, and the MTT assay performed as described in Materials & Methods. Lysates dissolved with DMSO were analyzed by monitoring absorbance at 560 and 670 nm. Four independent replicates were performed for each experiment (*p* value; * < 0.01, ** < 0.005, *** < 0.001).

Transport internalized EGFR to lysosomal compartments for degradation leads to shutdown of EGFR signaling. The downstream events of EGFR signaling involve activation of the Ras/MAPK and PI3K/Akt pathways, which generally promote cell survival and proliferation. We analyzed EGFR retention within cells during the period of endocytosis via immunoblot analysis (Fig. 6A). Gradual degradation of EGFR during the endocytic process culminated in the disappearance of EGFR after 10 min in control cells. However, enhanced stability of EGFR in SPIN90 knockdown cells was indicated by its presence, even after 30 min. ERK activity was proportional to the stability of EGFR, consistent with the ability of EGFR-mediated Ras/MAPK to activate ERK, which is reflected by its phosphorylation state. SPIN90 knockdown cells showed prolonged and stronger ERK activation, compared with the phospho-ERK signal in control cells (Fig 6A). In addition, PI3K activity for cell proliferation was also examined by the detection of phosphorylated Akt in SPIN90 knockdown cells. More phosphorylated Akt (active Akt) was observed in SPIN90 knockdown cells respectively (Fig. 6A d-e). To confirm the physiological effect of SPIN90, we examined cell proliferation through ERK signaling via detection of cyclin D1 (Fig. 6B). Cyclin proteins are generally expressed at specific points during the cell cycle. For example, cyclin D proteins, which are important for progression from the G1 phase to S phase, are upregulated upon EGF stimulation [30], [31], [32], [33]. We observed increased levels of cyclin D1 up to 36 h after EGF stimulation (Fig. 6B). Increased cyclin D1 levels were sustained, even at 36 h after EGF stimulation, in SPIN90 knockdown cells, compared with control cells, indicating prolonged endosomal retention of EGFR. Cell proliferation is reflected by cell numbers assessed with the MTT assay (Fig. 6C). Treatment with PD98059 together with EGF inhibited cell proliferation associated with knockdown of SPIN90. This result indicates that ERK activation by endosomal EGFR resulting from delayed endocytosis in SPIN90 knockdown cells induces physiological effects, such as cell proliferation.

## Discussion

Endocytosis of EGFR triggered by the binding of EGF continues during the maturation of early endosomes into either late endosomes or lysosomes, resulting in a dramatic decrease in cell surface EGFR and total cellular EGFR as part of the mechanism of ligand-induced receptor downregulation [34]. While the *EGFR* mRNA level was indistinguishable from that in control cells, the observed increase in cell surface area and total amount of EGFR (Figs. 1 and 4) and enhanced stability of EGFR, relative to control cells (Fig. 5), are suggestive of defective EGFR endocytosis in SPIN90 knockdown cells. The current study was aimed at investigating the role of SPIN90 in EGFR endocytosis.

Molecules internalized through receptor recognition are wrapped in membrane lipid and coated with clathrin prior to delivery to early endosomes for further processing [35]. Several endocytic regulators associated with docking/fusion and vesicle trafficking to early endosomes (such as SNAREs, Rab and effector proteins) are considered critical for the normal progression of endocytosis [36]. The autophagosome membrane nucleating protein, Atg14L, interacting with Snapin (a fusogenic SNARE effector protein), facilitates endosome maturation. However, knockdown of Atg14L significantly disrupts the late stage of endocytic trafficking, as evident from delayed degradation of internalized surface receptors [37]. The EGFR mutant unable to bind Grb2 does not efficiently enter coated pits, and Grb2 knockdown cells display dramatically reduced EGFR endocytosis [38]. In addition, depletion of Annexin A2 is reported to lead to delayed EGFR endocytosis and enhanced EGF-induced cell migration [39].

The early endosome targeting ability of TR-EGF-containing vesicles (trafficking) and the number and size of EEA1-labeled endosomes (vesicle/endosome fusion) were dramatically reduced in SPIN90 knockdown cells, indicating that this genetic modification of cells abrogates endocytosis of EGFR (Figs. 2 and 3). Visualization of EGFR with immunogold staining and electron microscopy showed decreased abundance of EGFR and EGFR-containing endosome numbers during the periods of EGF stimulation in SPIN90 knockdown cells. These results clearly reveal endocytic defects in SPIN90 knockdown cells. Moreover, whereas EGFR in control cells was detected in endosomes located far from the plasma membrane, that in SPIN90 knockdown cells was localized near the plasma membrane.

As Rab5 is a regulator of early endosome dynamics with major functions in vesicle formation and trafficking to early endosomes [28], [40], we used CFP-tagged wild-type Rab5 to interpret the effect of SPIN90 in EGFR trafficking with endosome formation. Full-length SPIN90 overexpression generated doughnut-like enlarged Rab5 endosomes, which appeared different from the large spots observed in vector-expressing cells, indicative of enhanced endosome fusion (Fig. 4A). Overexpression of each SPIN90 variants revealed that the SH3 and PR domains in the N-terminal region of SPIN90, as well as the C-terminal CC domain are important for endosome formation and movement (Fig. 4B). Small endosomes resulting from either from the formation of immature vesicles or failure of endosome fusion were observed near the surfaces of cells overexpressing the N-terminal, C-terminal, SH3, PRD, and CC regions of SPIN90. The effects of overexpression of SH3 and PRD are possibly due to inhibition of interactions with dynamin I and/or syndapin I, which may account for defects in endosomal morphology and movement. Data obtained from cells overexpressing SPIN90 CC indicate that this domain participates in vesicle formation and movement through interactions with additional endocytic partners. The failure of immature endosomes to traffic with overexpressed SPIN90 segments implies that SPIN90 is closely associated with endosome formation and trafficking during the early stages of endocytosis.

We speculated that the delay in trafficking of EGFR-containing vesicles to early endosomes, which eventually become lysosomes, in SPIN90 knockdown cells triggers physiological effects. For example, cell migration, which is controlled by endocytosis of PDGF, is mediated via PDGF receptor interactions with Grb2, DOCK4 and Dynamin2 [41]. In addition, translocation of internalized receptors to the nucleus facilitates receptor-mediated regulation of the transcription of specific target genes through direct binding to transcription factors or general co-regulators, which control DNA replication, DNA repair, and RNA metabolism. Nuclear localization of CCN, EGF/EGFR and FGF/FGFR is often detected in cancer cells, in correlation with tumor progression [42]. However, EGFR analysis during active endocytosis has shown that endosomal EGFR associates with many downstream effectors during MAPK and Akt signaling [43], [44], [45]. A study with a *rab-7* deletion mutant of *C. elegans* showed that defects in trafficking to lysosomes cause abnormal vulva development, which contributes to oncogenesis, reflecting that regulation of endocytic progression is closely involved in cell survival and proliferation [15], [46], [47]. In the current study, increased EGFR stability in SPIN90 knockdown cells was associated with endocytic defects (Fig. 6A). Under serum-starved conditions, the basal level of EGFR was elevated in SPIN90 knockdown cells, and EGFR stability was prolonged, relative to that in control cells. Consistent with previous reports, ERK activation was enhanced (stronger and prolonged) in SPIN90 knockdown cells, relative to control cells, given that activated endosomal EGFR stimulates the activity of downstream molecules. Cyclins targeted by EGFR signaling control mitotic events through distinctive patterns of expression and degradation. Among these, cyclin D1 is required for the G1-to-S transition. Earlier transcriptional profiling studies have shown that cyclin D1 is a critical downstream effector of EGFR signaling [48] and EGF-stimulated cyclin D1 expression is closely associated with tumor development caused by enhanced cell survival and proliferation [49]. The role in cell proliferation was examined through continuous monitoring of EGF signaling with the MTT assay using SPIN90 knockdown cells, either in the presence or absence of the ERK inhibitor, PD98059, and comparing it to EGF signaling in control cells (Fig. 5C). In serum-depleted culture conditions, cell proliferation was not distinguishable between control and SPIN90 knockdown cells, whereas EGF dramatically enhanced cell division in SPIN90 knockdown cells. Treatment with PD98059 efficiently blocked ERK activation, which suppressed proliferation in SPIN90 knockdown cells.

Our data collectively suggest that SPIN90 is a novel regulator of EGFR endocytosis, closely related with vesicle movement and formation in the early stages of the endocytic process. Increased endosomal EGFR due to delayed endocytosis enhances physiological effects, such as cell proliferation. Further studies focusing on domain mapping of SPIN90 in association with endocytic proteins are required to validate the role of SPIN90 in EGFR endocytosis and elucidate its function in endosome fusion and trafficking.
